# Quantitative variations in texture analysis features dependent on MRI scanning parameters: A phantom model

**DOI:** 10.1002/acm2.12482

**Published:** 2018-10-27

**Authors:** Karen Buch, Hirofumi Kuno, Muhammad M. Qureshi, Baojun Li, Osamu Sakai

**Affiliations:** ^1^ Department of Radiology Boston Medical Center Boston University School of Medicine Boston MA USA; ^2^ Department of Diagnostic Radiology National Cancer Center Hospital East Kashiwa Chiba Japan; ^3^ Departments of Radiology and Radiation Oncology Boston Medical Center Boston University School of Medicine Boston MA USA; ^4^ Departments of Radiology, Otolaryngology – Head and Neck Surgery, and Radiation Oncology Boston Medical Center Boston University School of Medicine Boston MA USA

**Keywords:** phantom, quantitative MRI, texture analysis

## Abstract

**Objectives:**

To evaluate the influence of MRI scanning parameters on texture analysis features.

**Methods:**

Publicly available data from the Reference Image Database to Evaluate Therapy Response (RIDER) project sponsored by The Cancer Imaging Archive included MRIs on a phantom comprised of 18 25‐mm doped, gel‐filled tubes, and 1 20‐mm tube containing 0.25 mM Gd‐DTPA (EuroSpinII Test Object5, Diagnostic Sonar, Ltd, West Lothian, Scotland). MRIs performed on a 1.5 T GE HD, 1.5 T Siemens Espree (VB13), or 3.0 T GE HD with TwinSpeed gradients with an eight‐channel head coil included T1WIs with multiple flip angles (flip‐angle = 2,5,10,15,20,25,30), TR/TE = 4.09–5.47/0.90–1.35 ms, NEX = 1 and DCE with 30° flip‐angle, TR/TE=4.09–5.47/0.90–1.35, and NEX = 1,4. DICOM data were imported into an in‐house developed texture analysis program which extracted 41‐texture features including histogram, gray‐level co‐occurrence matrix (GLCM), and gray‐level run‐length (GLRL). Two‐tailed *t* tests, corrected for multiple comparisons (*Q* values) were calculated to compare changes in texture features with variations in MRI scanning parameters (magnet strength, flip‐angle, number of excitations (NEX), scanner platform).

**Results:**

Significant differences were seen in histogram features (mean, median, standard deviation, range) with variations in NEX (*Q *= 0.003–0.045) and scanner platform (*Q *< 0.0001), GLCM features (entropy, contrast, energy, and homogeneity) with NEX (*Q *= 0.001–0.018) and scanner platform (*Q *< 0.0001), GLRL features (long‐run emphasis, high gray‐level run emphasis, high gray‐level emphasis) with magnet strength (*Q *= 0.0003), NEX (*Q *= 0.003–0.022) and scanner platform (*Q *< 0.0001).

**Conclusion:**

Significant differences were seen in many texture features with variations in MRI acquisition emphasizing the need for standardized MRI technique.

**Abbreviations**
GLCM: gray‐level co‐occurrence matrix
GLGM: gray‐level gradient matrix
GLN: gray‐level nonuniformity
GLRL: gray‐level run‐length
NEX: number of excitations
RLN: run‐length nonuniformity
SRE: short‐run emphasis
SRLGE: short‐run low gray‐level emphasis

## 1 INTRODUCTION

Increasing radiology research efforts have been focused on the investigation of potential genotype‐phenotype relationships of tumor biology and behavior, often referred to as radiomics. Radiomics has been employed in an effort to identify distinct behavioral subtypes of tumors based on their imaging appearance, with the ultimate hope of predicting tumor prognosis and treatment response. This field of radiomics often uses quantitative post‐processing techniques, known as a texture analysis, to identify unique pixel intensity patterns, or textures, within a tumor lesion based on its imaging appearance (e.g., CT, MRI, ultrasound, etc.).

Texture analysis describes the patterns of pixel intensity variations within an image calculated by a series of mathematical algorithms.^1^ Numerous texture analysis features have been described in the literature and defined in the work of Haralick et al.^1^ The use of a texture analysis applied to imaging studies including CT and MRI have been previously performed for the evaluation of multiple nonneoplastic disorders including the evaluation for mesial temporal sclerosis on MRI,^2^ evaluation of intervertebral disc disease on MRI,^3^ evaluation of hepatic fibrosis on both CT and MRI,^4^, ^5^, ^6^, ^7^, ^8^ evaluation of subchondral bone on MRI.^9^ Prior oncologic studies have also employed texture analyses to evaluate specific tumor features including the assessment of HPV status of oropharyngeal squamous cell carcinomas,^8^ prognosis of head and neck neoplasms,^10^, ^11^, ^12^ classification of gastric and colorectal tumors on CT,^13^, ^14^, ^15^ genomic mapping and predictive marker identification of gliomas on MRI,^16^, ^17^, ^18^, ^19^ the identification of potentially prognostic predictors in lung cancer,^20^, ^21^ evaluation of genitourinary neoplasms on both CT and MRI,^22^, ^23^, ^24^, ^25^ and for the radiomic classifications of breast carcinoma subtypes.^26^, ^27^, ^28^

In an effort to study specific patterns of tumor biology correlating with different imaging appearances, multi‐institutional centers have worked toward pooling resources to make publicly available cancer imaging databases, such as The Cancer Imaging Archive (TCIA) and The Cancer Genomic Atlas (TCGA), to help facilitate research efforts in the arena of tumor genotype‐phenotype analyses.^16^, ^26^, ^28^ Prior research studies have used a radiomics approach for investigating prostate cancer radiotherapy responses,^25^ responsiveness of neoadjuvant chemotherapy in breast cancer,^28^ and prognostic predictions of advanced nasopharyngeal carcinoma.^12^ However, larger studies and systematic reviews on radiomics have noted methodological variations as a source of difficulty precluding an accurate and collective interpretation of data.^11^, ^29^, ^30^

Based on our knowledge of how changes in the CT scanning parameters varies texture analysis features^30^, as well as preliminary studies investigating the sensitivity of texture features to variations in MRI technique,^29^, ^31^, ^32^, ^33^ we could similarly deduce that changes in MRI scanning parameters such as differences in magnet strength and scanner platform could also influence texture analysis features. Thus, the purpose of this study was to evaluate and quantify changes in MRI sequence parameters may have on texture analysis features using a simple, nonanatomic phantom model.

## 2 MATERIALS AND METHODS

This study employed the use of a phantom for all image acquisitions, precluding the requirement for IRB approval.

### 2.A Phantom development and MR imaging techniques

The construction of the phantom, and scan data of serial MRI scans of this phantom are publicly available as part of the Reference Image Database to Evaluate Therapy Response (RIDER) at The Cancer Imaging Archive (TCIA).^34^ The original DICOM datasets and scan data on the RIDER phantom are available for public use in an effort to generate an initial consensus on how to harmonize the data collection and analysis for quantitative imaging methods applied to the measurement of drug and/or radiation treatment response.^35^

**Figure 1:**
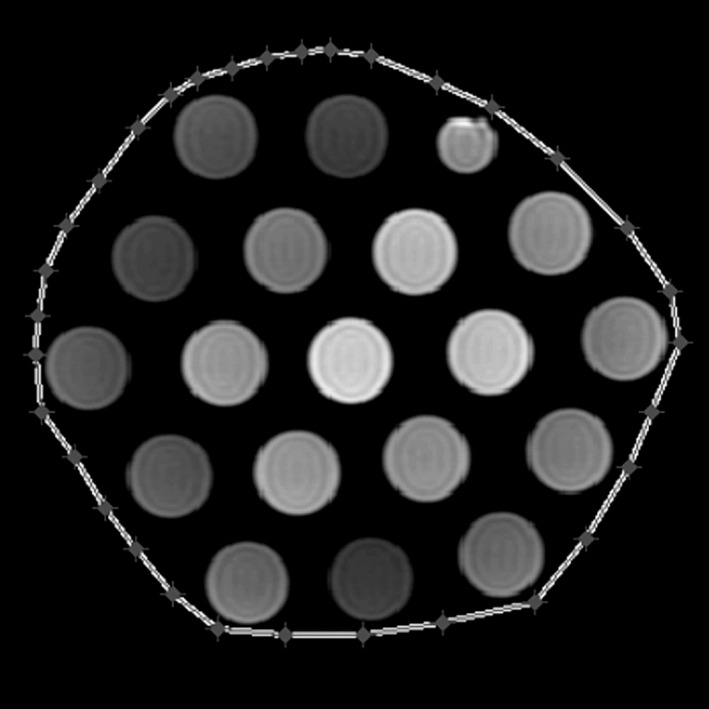
A cross‐sectional T1 weighted DICOM image through the nonanatomic phantom with a peripheral contour created within our in‐house developed MatLab platform. The phantom was composed of 18 dope‐filled gel tubes and a single tube filled with 0.25 mM of Gd‐DTPA.

The nonanatomic phantom used in the RIDER database was comprised of 18 25‐mm doped gel‐filled tubes, and a single 20‐mm tube containing 0.25 mM GdDTPA (EuroSpin II Test Object 5, Diagnostic Sonar, Ltd, West Lothian, Scotland),^34^ as shown in Fig 1.

All MRI examinations were performed at MD Anderson on either a 1.5 T GE HD, a 1.5 T Siemens Espree (VB13), or a 3.0 T GE HD with TwinSpeed gradients. An eight‐channel head coil was used for all scans.

Acquired scans included a T1‐weighted image using multiple flip angles, three‐dimensional Fast Spoiled Gradient Recalled Echo Sequence with flip angles = 2,5,10,15,20,25,30, a TR/TE = 4.09–6.469/0.90–1.35 ms, receiver bandwidth = ±31.25 kHz, 256 × 192 matrix, NEX = 1, slice thickness = 5 mm, and a 24 × 19 cm field of view.

A DCE acquisition was also performed using a three‐dimensional Fast Spoiled Gradient Recalled Echo Sequence with 30‐degree flip angle, a TR/TE = 4.09–5.47/0.90–1.35, receiver bandwidth = ±31.25 kHz, and NEX = 1,4, slice thickness = 5 mm, 256 × 160 matrix, and a 24 × 19 cm field of view.

### 2.B DICOM segmentation and texture analysis

Original DICOM data sets were downloaded and then imported into in‐house developed MATLAB (MathWorks, Natick, MA) texture analysis software to calculate texture analysis features. The texture analysis software was developed by the co‐author (BL) and the use of this texture analysis program has been previously reported in the literature.^7^, ^8^, ^30^ Image segmentation of phantom was performed manually by an experienced radiologist (co‐author HK), using the same geometric boundaries and a uniform contour volume for each dataset in an effort to reduce potential variation related to the manual segmentation process. The entirety of the phantom was contoured including each of the doped gel‐filled tubes, the gadolinium filled tube, as well as the negative space between in the inserts. A correction for spatial inhomogeneity was not applied. Prior to the texture analysis, the contoured images were preprocessed (or corrected) which consisted of the following steps: (a) partial volume artifact correction, and (b) global grayscale normalization. These steps are described in the work by Li et al.^7^ In brief, to correct for partial volume artifact, an optimal thresholding algorithm was applied using an iterative optimal thresholding algorithm.^36^ This method assumes all image pixels are from two probability distributions (e.g., structure of interest and the dark background) and attempts to find the gray‐level threshold corresponding to the minimum probability between the maxima of the two distributions, which results in minimal segmentation error. To find the optimal threshold, this algorithm was applied iteratively (usually four to ten iterations were sufficient), updating the threshold in each iteration from the weighted sum of the two distributions. For global grayscale normalization, the images were corrected by the mean and standard deviation to minimize the overall grayscale variation across images, similar to that described in the work of Collewet et al.^33^ The correction was applied to the entire image. The mean gray value of each corrected image was set to 250 and the standard deviation to 30.

In total, 41 texture features, including 12 histogram features, five gray‐level co‐occurrence matrix (GLCM) features, 11 gray‐level run‐length (GLRL) features, four gray‐level gradient matrix (GLGM) features, and nine Laws features, were calculated and averaged over the contoured images of each dataset. Numerous texture analysis equations have been defined and developed. Only a subset of 41 texture features were employed in this study based on our prior work, and based on the popularity of reported texture features in the radiomics literature.^7^, ^8^, ^30^

The use of our in‐house developed MATLAB program and the specific details of the texture analysis features calculated by this program have been previously published.^7^, ^8^, ^30^ A full description of the mathematical equations is described in the work by Haralick et al.^1^ and Tang el al.^37^ GLCM features, in contrast to histogram features, are highly spatially dependent. In this study, the GLCM texture features were calculated using only directly adjacent pixels for simplicity. Horizontal, 45°, vertical, and 135° directions were averaged together to eliminate any directional dependence. The following GLCM features proposed by Haralick et al.^1^ were tested:

(1) $$\text{Contrast}\;=\sum_{i,j}{\left|i-j\right|}^{2}p\left(i,j\right)$$

(2) $$\text{Correlation}\;=\sum_{i,j}\frac{\left(i-\mu_{i}\right)\;\left(j-\mu_{j}\right)p\left(i,j\right)}{\sigma_{i}\sigma_{j}}$$

(3) $$\text{Angular Second Moment (ASM)}\;=\sum_{i,j}p{\left(i,j\right)}^{2}$$

(4) $$\text{Homogeneity}\;=\sum_{i,j}\frac{p(i,j)}{1+|i-j|}$$

(5) $$\text{Entropy}\;=\sum_{i,j}\ln\left(p(i,j)\right)p\left(i,j\right)$$

where (*i*,* j*) represents the (*i*,* j)* value of the GLCM.

GLRL matrices were used as these texture features provide additional insights into spatial dependence18. The same directions considered for the calculation of the GLCM features, were averaged for the GLRL matrix features. The features explored included equations utilizing short‐run emphasis (SRE), long‐run emphasis (LRE), gray‐level nonuniformity (GLN), run‐length nonuniformity (RLN), run percentage (RP), low gray‐level run emphasis (LGRE), high gray‐level run emphasis (HGRE), short‐run low gray‐level emphasis (SRLGE), short‐run high gray‐level emphasis (SRHGE), long‐run low gray‐level emphasis (LRLGE), and long‐run high gray‐level emphasis (LRHGE), defined as follows:

(6) $$\text{SRE}\;=\frac{1}{n_{r}}\sum_{i,j}\frac{p(i,j)}{j^{2}}$$

(7) $$\text{LRE}\;=\frac{1}{n_{r}}\sum_{i,j}p(i,j)j^{2}$$

(8) $$\text{GLN}\;=\frac{1}{n_{r}}\sum_{i}{\left(\sum_{j}p\left(i,j\right)\right)}^{2}$$

(9) $$\text{RLN}=\frac{1}{n_{r}}\sum_{j}{\left(\sum_{i}p(i,j)\right)}^{2}$$

(10) $$\text{RP}=\frac{n_{r}}{n_{p}}$$

(11) $$\text{LGRE}=\frac{1}{n_{r}}\sum_{i,j}\frac{p(i,j)}{i^{2}}$$

(12) $$\text{HGRE}=\frac{1}{n_{r}}\sum_{i,j}p\left(i,j\right)i^{2}$$

(13) $$\text{SRLGE}=\frac{1}{n_{r}}\sum_{i,j}\frac{p(i,j)}{i^{2}j^{2}}$$

(14) $$\text{SRHGE}=\frac{1}{n_{r}}\sum_{i,j}\frac{p\left(i,j\right)i^{2}}{j^{2}}$$

(15) $$\text{LRLGE}=\frac{1}{n_{r}}\sum_{i,j}\frac{p\left(i,j\right)j^{2}}{i^{2}}$$

(16) $$\text{LRHGE}=\sum_{i,j}p\left(i,j\right)i^{2}j^{2}$$

where *p*(*i*,* j*) represents the (*i, j*) value of the GLRL matrix, nr is the total number of runs, and np is the total number of pixels.

GLGM features were also investigated to provide the histogram of the absolute gradient values in the interrogated region of interest. As a preprocessing step, the gradient of each pixel within the ROI was computed using a 3 × 3 neighborhood. The GLGM features mathematically summarize the gradient values of the pixels in the ROI and include mean, variance, skewness, and kurtosis.

### 2.C Statistical analysis

Serial MRI scans on the phantom were performed with variations in specific MRI scanning parameters. Multiple scan sequences are available in the RIDER dataset where a specific MRI scanning parameter is varied i.e., flip angle, while the remaining scanning parameters are held constant. We collated such scans where individual scanning parameters were sequentially varied in order to make the following assessments:

1. Assessment of Magnet Strength: T1‐weighted images performed on a 1.5 T GE Signa Excite compared to a T1‐weighted image performed on a 3 T GE Signa Excite with flip angle, TR/TE, number of excitations (NEX), echo train length, slice thickness, and matrix held constant
2. Assessment of Flip Angle: T1‐weighted images performed on a 1.5 T Siemens Espree with flip angle varying from 2, 5, 10, 15, 20, 25, and 30 degrees with the TRTE, NEX, echo train length, slice thickness, and matrix held constant.
3. Assessment of NEX: DCE images performed on a 1.5 T Siemens Espree with NEX either 1, or 4. The flip angle, TR/TE, slice thickness, and matrix were held constant
4. Assessment of Scanner Platform: DCE images were performed on a 1.5 T GE Signal Excite compared to a 1.5 T Siemens Espree with the flip angle, TR/TE, NEX, echo train length, slice thickness and matrix held constant.

For each of the four comparisons, a student's *t* test for assessing independent samples was used to evaluate variations in the 41 texture features based and was reported as a *P* value. To adjust for multiple comparisons, a false discovery rate (FDR) correction was performed and the FDR correction of the *P* values (termed *Q* values) were calculated in addition to raw *P* values using Benjamini and Hochberg method described in the literature.^38^ Statistical computations were performed using SAS 9.1.3 software (SAS Institute, Cary, NC).

## 3 RESULTS

Changes in texture analysis features based on variations in MR scanning parameters are shown in Tables 1, 2, 3, 4, and Table S1.

**Table 1:** Texture parameters: 1.5T vs 3T

	1.5T (*n* = 80)		3T (*n* = 61)		*P* value	*Q* value
	Mean	SD	Mean	SD		
Histogram						
Mean	247.2	2.2	246.8	2.4	0.225	0.298
Median	244.7	4.8	243.4	5.7	0.170	0.239
STD	30.6	4.5	31.8	5.7	0.184	0.251
Range	8.3	2.8	7.9	2.4	0.465	0.554
Geometric mean	248.2	0.49	248.0	0.61	0.142	0.220
Harmonic mean	246.4	0.90	246.2	1.1	0.164	0.238
2nd STD	2.9	1.0	2.7	0.84	0.336	0.432
STD5	3.1	1.1	2.9	0.96	0.159	0.238
STD9	3.2	1.0	3.2	0.93	0.892	0.934
4th moment	3069652.7	2076989.3	4128044.7	2905848.7	**0.018**	**0.045**
IQR	39.6	4.3	39.1	3.8	0.503	0.580
Entropy	7.2	0.31	7.2	0.25	0.702	0.790
GLCM						
Entropy	2.1	0.45	2.1	0.43	0.745	0.818
Contrast	16.9	6.5	18.9	6.6	0.075	0.143
Correlation	0.91	0.08	0.90	0.08	0.360	0.450
Energy	0.01	0.003	0.01	0.003	0.817	0.875
Homogeneity	0.60	0.05	0.59	0.04	0.940	0.940
GLRL						
SRE	0.09	0.03	0.10	0.04	0.054	0.128
LRE	0.09	0.04	0.10	0.04	0.065	0.133
GLN	0.09	0.03	0.10	0.04	0.090	0.153
RLN	0.09	0.04	0.10	0.04	0.063	0.133
RP	162.1	42.3	149.4	41.2	0.076	0.143
LGRE	159.5	43.3	147.1	42.5	0.092	0.153
HGRE	157.9	42.6	146.4	41.7	0.113	0.182
SRLGE	159.8	43.4	147.2	42.5	0.087	0.153
SRHGE	3052.4	1691.4	1833.1	626.2	**<0.0001**	**0.0003**
LRLGE	3661.3	2077.6	2100.8	755.5	**<0.0001**	**0.0003**
LRHGE	2647.5	1455.7	1568.1	532.6	**<0.0001**	**0.0003**
Law's features						
L1	162232.6	83374.1	252842.7	54759.9	**<0.0001**	**0.0003**
L2	18049.8	12630.7	30827.7	11995.7	**<0.0001**	**0.0003**
L3	6438.0	3814.3	10443.2	2874.5	**<0.0001**	**0.0003**
L4	33648.4	14046.5	49080.4	7890.0	**<0.0001**	**0.0003**
L5	6830.8	7083.6	13929.7	6922.4	**<0.0001**	**0.0003**
L6	5044.4	4893.9	9891.0	4395.4	**<0.0001**	**0.0003**
L7	3793.4	3264.1	7070.4	2912.7	**<0.0001**	**0.0003**
L8	10919.5	9724.9	20981.7	8112.5	**<0.0001**	**0.0003**
L9	15398.6	6675.3	22754.1	3790.9	**<0.0001**	**0.0003**
GLGM						
MGR	20.7	10.2	29.1	13.0	**<0.0001**	**0.0003**
VGR	16410.5	8816.7	24038.9	11063.9	**<0.0001**	**0.0003**
Skewness	7.7	2.0	6.5	1.9	**0.001**	**0.003**
Kurtosis	67.6	34.4	50.2	29.0	**0.002**	**0.005**
Mean skewness	0.64	0.59	0.72	0.59	0.468	0.554
Mean kurtosis	3.0	0.5	3.2	0.6	0.063	0.133
Mean laws	270470.4	141778.4	423628.7	100109.0	**<0.0001**	**0.0003**

Mean texture analysis features on a 1.5 T vs a 3.0 T scanner. n: number of contoured slices; STD: standard deviation; STD5: 5‐neighborhood standard deviation; STD9: 9‐neighborhood standard deviation; IQR: indicates interquartile range; GLCM: gray‐level co‐occurrence matrix; GLRL: gray‐level run length; SRLGE: short‐run low gray‐level emphasis; SRHGE: short‐run high gray‐level emphasis; GLGM: gray‐level gradient matrix; SRE: short‐run emphasis; LRE: long‐run emphasis; GLN: gray‐level nonuniformity; RLN: run‐length nonuniformity; RP: run percentage; LGRE: low gray‐level run emphasis; HGRE: high gray‐level run emphasis; SRLGE: short‐run low gray‐level emphasis; SRHGE: short‐run high gray‐level emphasis; LRLGE: long‐run low gray‐level emphasis; LRHGE: long‐run high gray‐level emphasis; MGR: mean gradients; VGR: variance of gradients.

Bold indicates statistically significant as determined with the two‐tailed *t* test and false detection analyses (*Q* < 0.05).

**Table 2:** Texture parameters: mean by flip angle

	2 (*n* = 36)	5 (*n* = 36)	10 (*n* = 36)	15 (*n* = 36)	20 (*n* = 36)	25 (*n* = 36)	30 (*n* = 44)	*P*‐value	*Q*‐value
Histogram									
Mean	248.0	246.6	246.4	247.3	247.1	247.3	247.5	0.106	0.227
Median	247.2	243.8	241.4	245.6	247.4	246.5	244.8	**<0.0001**	**0.002**
STD	27.0	29.5	30.3	28.5	29.2	28.2	27.7	0.081	0.220
Range	10.2	9.3	9.5	9.7	10.2	10.1	10.0	0.693	0.958
Geometric mean	248.6	248.3	248.2	248.4	248.3	248.4	248.5	0.104	0.227
Harmonic mean	247.2	246.7	246.6	246.9	246.8	247.0	247.1	0.102	0.227
2nd STD	3.4	3.1	3.2	3.3	3.5	3.4	3.4	0.697	0.958
STD5	4.3	4.0	4.1	4.1	4.3	4.3	4.2	0.826	0.958
STD9	5.1	4.9	5.1	5.0	5.3	5.2	5.2	0.946	0.992
4th moment	2166675.3	2993396.8	3359004.4	2519117.0	3053199.6	2557749.2	2269343.1	0.298	0.559
IQR	39.0	41.3	41.5	40.2	40.2	39.9	39.4	0.362	0.603
Entropy	7.5	7.4	7.3	7.4	7.0	7.0	7.1	**<0.0001**	**0.002**
GLCM									
Entropy	2.7	2.3	2.1	2.4	2.3	2.4	2.4	0.021	0.118
Contrast	18.0	14.3	12.7	16.4	17.5	17.5	18.3	**0.001**	**0.011**
Correlation	0.92	0.94	0.95	0.93	0.92	0.92	0.92	0.019	0.118
Energy	0.01	0.01	0.01	0.01	0.01	0.01	0.01	0.087	0.220
Homogeneity	0.53	0.56	0.57	0.55	0.54	0.54	0.54	0.036	0.135
GLRL									
SRE	0.09	0.11	0.13	0.11	0.11	0.11	0.11	0.756	0.958
LRE	0.09	0.11	0.13	0.11	0.11	0.11	0.11	0.813	0.958
GLN	0.09	0.11	0.13	0.11	0.11	0.11	0.10	0.861	0.969
RLN	0.09	0.11	0.13	0.11	0.11	0.11	0.11	0.801	0.958
RP	174.7	149.3	134.2	164.2	172.6	172.8	180.9	0.988	0.992
LGRE	174.7	149.0	133.5	163.9	172.1	171.7	179.6	0.986	0.992
HGRE	178.1	153.7	137.9	167.8	176.3	175.6	184.2	0.989	0.992
SRLGE	173.5	147.8	132.6	162.7	171.1	171.2	179.2	0.992	0.992
SRHGE	2917.7	2167.3	1952.6	2589.0	2569.0	2592.7	2549.7	0.830	0.958
LRLGE	3424.4	2631.8	2429.1	3029.8	3003.8	3049.7	2988.9	0.806	0.958
LRHGE	2910.0	2195.3	2006.0	2568.6	2571.9	2606.3	2569.7	0.656	0.958
Law's features									
L1	192705.6	192761.2	192439.2	193311.5	195378.8	194563.7	193770.3	0.733	0.958
L2	16489.5	16373.4	16323.5	16576.5	17381.4	17285.1	16808.4	**0.001**	**0.011**
L3	6162.2	6141.1	6255.8	6155.8	6387.6	6363.1	6304.5	0.073	0.219
L4	38082.9	37979.5	37990.6	38104.2	38882.5	38666.9	38384.9	0.424	0.681
L5	4816.5	4812.1	4769.3	4869.8	5128.8	5112.0	4929.7	0.034	0.135
L6	4024.3	4032.9	3989.2	4066.0	4269.3	4269.7	4111.7	0.027	0.135
L7	3002.0	3000.4	3081.4	3010.1	3136.7	3145.4	3091.9	0.035	0.135
L8	9396.0	9481.2	9217.9	9434.4	10117.2	10063.3	9574.5	**0.010**	**0.090**
L9	18404.7	18234.2	18211.3	18321.4	18878.1	18750.6	18650.3	0.199	0.407
GLGM									
MGR	14.5	13.4	13.5	14.1	13.1	13.4	12.7	0.329	0.569
VGR	11028.3	10337.9	10427.8	10781.4	9984.6	10181.8	9640.4	0.213	0.417
Skewness	8.5	8.8	8.7	8.7	9.1	9.0	9.3	0.088	0.220
Kurtosis	78.1	83.2	82.1	81.4	89.2	88.6	94.6	0.045	0.151
Mean skewness	0.46	0.80	0.92	0.62	0.60	0.58	0.54	0.020	0.118
Mean kurtosis	2.8	3.1	3.4	3.0	3.2	3.1	3.1	0.047	0.151
Mean laws	300502.0	300676.9	300393.7	301260.7	307050.4	305634.3	303027.0	0.320	0.569

Mean texture analysis features variation with changes in flip angle. n: number of contoured slices; STD: standard deviation; STD5: 5‐neighborhood standard deviation; STD9: 9‐neighborhood standard deviation; IQR: indicates interquartile range; GLCM: gray‐level co‐occurrence matrix; GLRL: gray‐level run length; SRLGE: short‐run low gray‐level emphasis; SRHGE: short‐run high gray‐level emphasis; GLGM: gray‐level gradient matrix; SRE: short‐run emphasis; LRE: long‐run emphasis; GLN: gray‐level nonuniformity; RLN: run‐length nonuniformity; RP: run percentage; LGRE: low gray‐level run emphasis; HGRE: high gray‐level run emphasis; SRLGE: short‐run low gray‐level emphasis; SRHGE: short‐run high gray‐level emphasis; LRLGE: long‐run low gray‐level emphasis; LRHGE: long‐run high gray‐level emphasis; MGR: mean gradients; VGR: variance of gradients.

Bold indicates statistically significant as determined with the two‐tailed *t*‐test and false detection analyses (*Q* < 0.05).

**Table 3:** Texture parameters: number of excitations 1 vs 4

	1 (*n* = 36)		4 (*n* = 44)		*P*‐value	*Q*‐value
	Mean	SD	Mean	SD		
Histogram						
Mean	249.6	2.3	247.5	2.8	**0.001**	**0.003**
Median	249.5	6.0	244.8	6.8	**0.002**	**0.005**
STD	24.3	4.2	27.7	4.9	**0.002**	**0.005**
Range	11.4	2.3	10.0	2.7	**0.013**	**0.022**
Geometric mean	248.8	0.38	248.5	0.45	**0.002**	**0.005**
Harmonic mean	247.6	0.69	247.1	0.83	**0.003**	**0.006**
2nd STD	3.9	0.78	3.4	0.93	**0.013**	**0.022**
STD5	4.8	1.0	4.2	1.1	**0.028**	**0.045**
STD9	5.7	1.3	5.2	1.4	0.107	0.146
4th moment	1257285.5	1220668.6	2269343.1	1656035.4	**0.003**	**0.006**
IQR	35.1	5.0	39.4	6.2	**0.001**	**0.003**
Entropy	7.2	0.48	7.1	0.29	0.413	0.502
GLCM						
Entropy	3.0	0.61	2.4	0.69	**0.0001**	**0.001**
Contrast	23.1	7.7	18.3	8.5	**0.010**	**0.018**
Correlation	0.89	0.04	0.92	0.05	**0.005**	**0.009**
Energy	0.005	0.002	0.008	0.004	**<0.0001**	**0.001**
Homogeneity	0.49	0.06	0.54	0.07	**0.002**	**0.005**
GLRL						
SRE	0.07	0.04	0.11	0.05	**0.001**	**0.003**
LRE	0.07	0.04	0.11	0.05	**0.0004**	**0.002**
GLN	0.07	0.04	0.10	0.05	**0.001**	**0.003**
RLN	0.07	0.04	0.11	0.05	**0.001**	**0.003**
RP	211.7	63.2	180.9	66.2	**0.038**	**0.055**
LGRE	212.3	63.5	179.6	66.9	**0.029**	**0.045**
HGRE	214.4	62.7	184.2	65.3	0.040	0.056
SRLGE	212.1	64.1	179.2	67.8	**0.030**	**0.045**
SRHGE	3842.0	1316.4	2549.7	1735.8	**0.0004**	**0.002**
LRLGE	4431.0	1453.1	2988.9	1914.6	**0.0004**	**0.002**
LRHGE	3845.2	1318.9	2569.7	1738.3	**0.001**	**0.003**
Law's features						
L1	194138.1	8172.2	193770.3	8258.3	0.843	0.843
L2	17098.3	1319.8	16808.4	1211.3	0.310	0.388
L3	6323.2	406.3	6304.5	380.0	0.832	0.843
L4	38727.6	2029.3	38384.9	2147.6	0.469	0.555
L5	5002.1	517.8	4929.7	427.0	0.495	0.571
L6	4142.0	409.5	4111.7	308.3	0.707	0.758
L7	3072.6	210.6	3091.9	170.2	0.651	0.715
L8	9678.2	1135.5	9574.5	872.1	0.645	0.715
L9	18972.1	1287.3	18650.3	1373.0	0.287	0.369
GLGM						
MGR	16.2	1.9	12.7	4.0	**<0.0001**	**0.001**
VGR	12135.7	1227.1	9640.4	2842.2	**<0.0001**	**0.001**
Skewness	7.9	0.5	9.3	1.6	**<0.0001**	**0.001**
Kurtosis	67.2	9.0	94.6	32.2	**<0.0001**	**0.001**
Mean skewness	0.03	0.64	0.54	0.75	**0.002**	**0.005**
Mean kurtosis	2.7	0.63	3.1	0.58	**0.004**	**0.008**
Mean laws	303992.7	14617.7	303027.0	14279.6	0.767	0.803

Mean texture analysis features with variations in number of excitations (NEX). n: number of contoured slices; STD: standard deviation; STD5: 5‐neighborhood standard deviation; STD9: 9‐neighborhood standard deviation; IQR: indicates interquartile range; GLCM: gray‐level co‐occurrence matrix; GLRL: gray‐level run length; SRLGE: short‐run low gray‐level emphasis; SRHGE: short‐run high gray‐level emphasis; GLGM: gray‐level gradient matrix; SRE: short‐run emphasis; LRE: long‐run emphasis; GLN: gray‐level nonuniformity; RLN: run‐length nonuniformity; RP: run percentage; LGRE: low gray‐level run emphasis; HGRE: high gray‐level run emphasis; SRLGE: short‐run low gray‐level emphasis; SRHGE: short‐run high gray‐level emphasis; LRLGE: long‐run low gray‐level emphasis; LRHGE: long‐run high gray‐level emphasis; MGR: mean gradients; VGR: variance of gradients.

Bold indicates statistically significant as determined with the two‐tailed *t*‐test and false detection analyses (*Q* < 0.05).

**Table 4:** Texture parameters: GE vs Siemens

	GE (*n* = 83)		Siemens (*n* = 36)		*P*‐value	*Q*‐value
	Mean	SD	Mean	SD		
Histogram						
Mean	245.9	1.8	249.6	2.3	**<0.0001**	**0.0001**
Median	242.0	4.5	249.5	6.0	**<0.0001**	**0.0001**
STD	32.5	5.2	24.3	4.2	**<0.0001**	**0.0001**
Range	9.2	1.4	11.4	2.3	**<0.0001**	**0.0001**
Geometric mean	248.0	0.56	248.8	0.38	**<0.0001**	**0.0001**
Harmonic mean	246.1	1.04	247.6	0.70	**<0.0001**	**0.0001**
2nd STD	3.2	0.48	3.9	0.78	**<0.0001**	**0.0001**
STD5	3.6	0.56	4.8	0.96	**<0.0001**	**0.0001**
STD9	3.5	0.54	5.7	1.3	**<0.0001**	**0.0001**
4th moment	4384199.1	2820540.2	1259420.0	1216812.6	**<0.0001**	**0.0001**
I QR	41.0	3.1	35.1	5.1	**<0.0001**	**0.0001**
Entropy	7.3	0.20	7.2	0.48	0.516	0.554
GLCM						
Entropy	2.2	0.39	3.0	0.61	**<0.0001**	**0.0001**
Contrast	16.7	4.5	23.2	7.7	**<0.0001**	**0.0001**
Correlation	0.93	0.04	0.89	0.04	**<0.0001**	**0.0001**
Energy	0.009	0.002	0.005	0.002	**<0.0001**	**0.0001**
Homogeneity	0.57	0.02	0.49	0.06	**<0.0001**	**0.0001**
GLRL						
SRE	0.11	0.03	0.07	0.04	**<0.0001**	**0.0001**
LRE	0.11	0.03	0.07	0.04	**<0.0001**	**0.0001**
GLN	0.12	0.03	0.07	0.04	**<0.0001**	**0.0001**
RLN	0.11	0.03	0.07	0.04	**<0.0001**	**0.0001**
RP	144.5	19.2	212.0	63.3	**<0.0001**	**0.0001**
LGRE	141.0	20.6	212.6	63.6	**<0.0001**	**0.0001**
HGRE	139.5	20.4	214.8	62.7	**<0.0001**	**0.0001**
SRLGE	140.6	20.5	212.5	64.2	**<0.0001**	**0.0001**
SRHGE	2037.7	592.1	3844.4	1321.0	**<0.0001**	**0.0001**
LRLGE	2421.5	660.9	4429.5	1460.6	**<0.0001**	**0.0001**
LRHGE	1753.3	466.9	3845.8	1323.4	**<0.0001**	**0.0001**
Law's features						
L1	186793.4	33134.5	194202.9	8099.2	0.189	0.236
L2	17662.7	8333.2	17104.7	1293.8	0.691	0.707
L3	7092.6	2150.3	6323.8	399.1	**0.036**	**0.049**
L4	39166.7	4904.5	38746.2	2016.0	0.621	0.650
L5	5836.4	4462.7	5002.2	524.1	0.267	0.316
L6	4712.7	3135.9	4141.0	407.6	0.279	0.322
L7	3724.9	2070.2	3072.0	206.5	0.062	0.082
L8	10812.2	5484.5	9691.9	1184.0	0.229	0.279
L9	17868.8	2408.8	18978.0	1267.0	**0.010**	**0.014**
GLGM						
MGR	14.1	8.9	16.2	1.9	0.166	0.213
VGR	11304.8	7622.5	12137.4	1222.2	0.517	0.554
Skewness	9.2	1.7	7.9	0.52	**<0.0001**	**0.0001**
Kurtosis	94.3	30.4	67.2	9.0	**<0.0001**	**0.0001**
Mean skewness	0.95	0.42	0.03	0.64	**<0.0001**	**0.0001**
Mean kurtosis	3.2	0.47	2.7	0.67	**<0.0001**	**0.0001**
Mean laws	301700.2	63339.7	304095.9	14624.5	0.823	0.823

Mean texture analysis features based on scanner platform: GE vs Siemens. STD: standard deviation; STD5: 5‐neighborhood standard deviation; STD9: 9‐neighborhood standard deviation; IQR: indicates interquartile range; GLCM: gray‐level co‐occurrence matrix; GLRL: gray‐level run length; SRLGE: short‐run low gray‐level emphasis; SRHGE: short‐run high gray‐level emphasis; GLGM: gray‐level gradient matrix; SRE: short‐run emphasis; LRE: long‐run emphasis; GLN: gray‐level nonuniformity; RLN: run‐length nonuniformity; RP: run percentage; LGRE: low gray‐level run emphasis; HGRE: high gray‐level run emphasis; SRLGE: short‐run low gray‐level emphasis; SRHGE: short‐run high gray‐level emphasis; LRLGE: long‐run low gray‐level emphasis; LRHGE: long‐run high gray‐level emphasis; MGR: mean gradients; VGR: variance of gradients.

Bold indicates statistically significant as determined with the two‐tailed *t*‐test and false detection analyses (*Q* < 0.05).

### 3.A Assessment of magnetic strength

Variations in magnetic strength (1.5 T vs 3 T) resulting in changes in texture features are displayed in Table 1. No statistically significant differences were noticed in the histogram, or GLCM texture features. A few of the GLRL texture features including the short‐run high gray‐level emphasis (SRHGE), long‐run low gray‐level emphasis (LRLGE), and long‐run high gray‐level emphasis (LRHGE) demonstrated statistically significant differences (*Q *=* *0.0003), however, the remaining GLRL features did not demonstrate a significant difference. All Law's features demonstrated statistically significant differences (*Q *=* *0.0003), and all of the GLGM features, with the exception of mean skewness and mean kurtosis (*Q *=* *0.554, and 0.133, respectively), demonstrated statistically significant differences (*Q *=* *0.0003–0.005).

### 3.B Assessment of flip angle

Variations in flip angles produced variations in texture analysis features, as shown in Table 2 and Table S1. Only two histogram features, median and entropy, demonstrated statistically significant differences with changes in flip angle (*Q *=* *0.002, each). Similarly, only the GLCM feature, contrast, demonstrated a statistically significant difference related to changes in the flip angle (*Q *=* *0.011). No statistically significant difference in the GLRL features, Law's features, or GLGM features with variations in flip angle.

### 3.C Assessment of NEX

Changes in NEX (1 vs 4) produced variations in texture analysis features as shown in Table 3. All histogram texture features, with the exception of neighborhood standard deviation (STD9) (*Q *=* *0.146) and entropy (Q = 0.502), demonstrated statistically significant differences (*Q *=* *0.003–0.045). All GLCM texture features demonstrated statistically significant differences with changes in NEX (*Q *=* *0.001–0.018). All GLRL texture features, with the exception of run percentage (RP) (*Q *=* *0.055) and high gray‐level run emphasis (HGRE) (*Q *=* *0.056), demonstrated statistically significant differences with changes in NEX (*Q *=* *0.002–0.045). None of the Law's features demonstrated statistically significant differences. All of the GLGM texture features demonstrated statistically significant differences with changes in NEX (*Q *=* *0.001–0.008).

### 3.D Assessment of scanner platform

Differences in scanner platform (GE vs Siemens) produced differences in the texture analysis features as shown in Table 4. All histogram features, except for entropy (*P *=* *0.554), demonstrated statistically significant differences (*Q *=* *0.0001). All GLCM and GLRL texture features demonstrated statistically significant differences with different scanner platforms (*Q *=* *0.0001). Only the Laws feature, L9, demonstrated a statistically significant difference (*Q *=* *0.014). All GLGM texture features demonstrated statistically significant differences (*Q *=* *0.0001), with the exception of mean gradients (MGR) (*Q *=* *0.236), variance of gradients (VGR) (*Q *=* *0.554), and mean Laws features (*Q *=* *0.823).

## 4 DISCUSSION

The results of this study demonstrate statistically significant differences in multiple texture analysis features (histogram, GLCM, GLRL, and GLGM) related to changes in several, specific MRI scan parameters such as magnet strength, flip angle, NEX, and scanner platform.

While multiple prior research studies have investigated the use of a texture analysis applied to MR images^2^, ^3^, ^4^, ^5^, ^6^, ^7^, ^9^, ^10^, ^12^, ^15^, ^16^, ^17^, ^18^, ^19^, ^39^ the underlying influence of MRI scan parameters on texture analysis features are not entirely understood. Furthermore, despite the increasing use of texture analysis in the field of radiology, a fundamental understanding of the histopathologic and biologic correlation between tissue and texture analysis features remains in its infancy.

In this study, we demonstrated statistically significant differences in the Law's features and several GLGM features with differences in magnet strength, while histogram, GLCM, and GLRL features were invariant of these changes in magnet strength. Differences in flip angle significantly influenced GLCM texture features and changes in NEX significantly influenced histogram, many GLRL, and GLGM texture features. The most substantial changes in texture analysis features were encountered with differences in MRI scanner platform (GE vs Siemens). Differences in the MRI scanner platform generated statistically significant differences in all categories of texture analysis features, except for the Laws features. Histogram features by in large measure the image signal‐to‐noise ratio (SNR), which is mostly a low frequency signal. In contradistinction, Laws features measure distinct features within an image such as edges and lines, which are predominately high frequency signals. Changes in both NEX and flip angle would only affect the SNR, but not the spatial resolution. We postulate that different scanner platforms (i.e., GE vs Siemens and 3 T vs 1.5 T) employ dramatically different image processing algorithms and this is the primary reason there is a statistical significance observed in Laws features. On the contrary, histogram features are less sensitive to changes in spatial resolution and are more sensitive to changes in SNR (e.g., NEX, flip angle, etc.)

Changes in NEX and flip angle may only affect image contrast and would not affect spatial resolution. We postulate that different scanner vendors, i.e., GE and Siemens, employ dramatically different image processing algorithms and this is the primary reason there is a statistical significance observed in Laws features. On the contrary, histogram features are less sensitive to changes in spatial resolution and are more sensitive to changes in contrast (e.g., NEX, flip angle, etc.).

Multiple prior studies have highlighted the potential promise and importance of using a texture analysis as a quantitative, post‐processing technique to evaluate subtle changes in pixel intensity which may not be evident to the human eye.^7^, ^8^, ^14^ These subtle patterns of pixel variation could potentially serve as a biomarker for lesion characterization, early disease detection, and prediction of lesional behavior.^6^, ^8^, ^20^ A recent prior study demonstrated a dependency of texture analysis features on variations in CT scanning parameters.^30^ The results of this study, build off those from the prior study examining how texture analysis features are influenced by MRI, in addition to CT acquisition parameters. This work highlights the importance of using standardized and rigorously controlled scanning protocol when conducting research utilizing a texture analysis. This current study expands upon prior studies published in the literature which previously investigated a limited set of MRI acquisition parameters and their influence on texture features.^31^, ^32^ The study performed by Mayerofer et al.^31^ investigated changes in TR/TE, sampling bandwidth, and number of acquisitions and the influence of these parameters on texture analysis features. Mayerofer et al., noted that changes in these features had a substantial impact on the sensitivity of the texture analysis features,^31^ however, this study examined a limited set of MRI scanning parameters which did not include a study of NEX, flip angle, magnet strength, and scanner platform (GE vs Siemens). This current study seeks to bridge the gap in knowledge investigating the influence these additional MRI scanning parameters have on certain texture analysis features.

The results of this study underscore the importance of understanding how texture analysis features are influenced by imaging acquisition parameters. The ability to distinguish changes in texture analysis features related to tissue biology and pathology vs effects related to technical differences in MRI scanning protocol is of paramount importance for designing future research investigations which will use a texture analysis.

There are several limitations to the current study. The first is that this was a study using a nonanatomic phantom with basic architecture variations in internal structure. The use of this phantom and associated scanner data was advantageous as an initial pilot investigation into the dependency texture features on MRI scanning parameters as the raw scanning data are publicly available for research efforts. The phantom used in this study has a well‐defined, well‐characterized, and simple internal geometric structure. We recognize that the simplicity of this phantom is a far reach from a phantom with anatomically relevant internal structure, but we feel that the simplicity of this nonanatomic phantom initially helps us to understand the results of this study and the effects the changes in MRI scanning parameters has on the texture features. Future research efforts will need to be conducted using a phantom with more anatomically relevant internal structure and with more complex internal components, perhaps with an internal composition mimicking that of fat, muscle, and bone. Additionally, a robustness analysis on real test‐retest data should be also performed, similar to the work of van Timmeren et al. for CT.^40^ A second limitation of this study is that only a discrete subset of MRI scanning parameters was investigated. This study was limited based on the information available in the RIDER dataset. We would have liked to investigate the influence additional scanning parameters such as slice thicknesses, matrix size, and differences in TR/TE have on these texture analysis features, however, this information was not available in the RIDER dataset. Future investigations on this topic will also be to examine a broader set of MRI scan parameters and evaluate the influence these parameters have on texture analysis features. Thirdly, this study investigated a limited set of 41 defined texture analysis features. There are hundreds of defined texture analysis features described in the literature. We sought to investigate a subset of 41 texture features which we have investigated in our previous works, and which we feel are most frequently reported in the radiomics literature.^7^, ^8^, ^30^ We recognize that this subset may have excluded additional texture features of interest. The inclusion of additional texture features in our in‐house developed texture analysis program will be addressed in future research endeavors. Lastly a limitation of this study, is the investigation of only GRE‐based MRI sequenced. Again, our investigation was limited to the information available in the RIDER dataset. We do recognize that investigating how texture analysis features are influenced by MRI scanning parameters on non‐GRE based sequences would be of great interest. Future investigations in this subject matter with an expanded analysis of additional texture analysis features are warranted.

## 5 CONCLUSION

Texture analysis represents an increasingly popular, post‐processing, quantitative evaluation technique that can potentially be used as an adjunct in diagnostic imaging, and as a possible imaging biomarker. The results of this study demonstrate that MRI acquisition parameters have a significant influence on specific texture analysis features. This work serves as a pilot study highlighting the importance of using a standardized and controlled MRI scanning protocol when using a texture analysis. Multi‐institutional research endeavors, or single institution endeavors using different MRI scanning platforms and scanning protocols should exercise caution when using texture analysis.

## CONFLICT OF INTEREST

No conflicts of interest.

## Supporting information


**Table S1.** Texture parameters: Standard deviation by flip angle
