# Supplementary material for: Quantitative variations in texture analysis features dependent on MRI scanning parameters: A phantom model
Source: J Appl Clin Med Phys. 2018 Oct 27;19(6):253–64. doi: 10.1002/acm2.12482 (PMC6236836; doi:10.1002/acm2.12482)
Supplement: Supplementary file 1 — Table S1. Texture parameters: Standard deviation by flip angle [file ACM2-19-253-s001.docx]

| **Supplement Table 1. Texture parameters: Standard deviation by flip angle** | | | | | | | | |
| --- | --- | --- | --- | --- | --- | --- | --- | --- |
|  | 2 (n=36) | 5 (n=36) | 10 (n=36) | 15 (n=36) | 20 (n=36) | 25 (n=36) | 30 (n=44) |  |
| Histogram |  |  |  |  |  |  |  |  |
| Mean | 2.5 | 2.0 | 1.7 | 2.4 | 3.0 | 2.8 | 2.8 |  |
| Median | 4.2 | 4.1 | 4.7 | 5.3 | 4.5 | 5.3 | 6.8 |  |
| STD | 5.2 | 4.6 | 4.4 | 5.0 | 5.8 | 5.2 | 4.9 |  |
| Range | 2.0 | 2.0 | 2.8 | 2.4 | 3.1 | 3.2 | 2.7 |  |
| Geometric mean | 0.51 | 0.46 | 0.50 | 0.47 | 0.55 | 0.50 | 0.45 |  |
| Harmonic mean | 0.94 | 0.85 | 0.92 | 0.87 | 1.0 | 0.91 | 0.83 |  |
| 2^nd^ STD | 0.69 | 0.70 | 1.0 | 0.84 | 1.1 | 1.1 | 0.93 |  |
| STD5 | 0.88 | 0.90 | 1.3 | 1.0 | 1.2 | 1.3 | 1.1 |  |
| STD9 | 1.3 | 1.4 | 1.9 | 1.3 | 1.5 | 1.6 | 1.4 |  |
| 4^th^ moment | 2611731.5 | 2707690.1 | 3749160.2 | 1697489.2 | 2124445.3 | 2024603.6 | 1656035.4 |  |
| IQR | 4.7 | 3.8 | 2.9 | 5.0 | 6.7 | 6.3 | 6.2 |  |
| Entropy | 0.31 | 0.27 | 0.22 | 0.31 | 0.47 | 0.48 | 0.29 |  |
| GLCM |  |  |  |  |  |  |  |  |
| Entropy | 0.71 | 0.59 | 0.40 | 0.77 | 0.87 | 0.78 | 0.69 |  |
| Contrast | 5.9 | 3.1 | 2.4 | 6.0 | 7.6 | 7.5 | 8.5 |  |
| Correlation | 0.03 | 0.02 | 0.02 | 0.03 | 0.05 | 0.05 | 0.05 |  |
| Energy | 0.004 | 0.004 | 0.002 | 0.01 | 0.01 | 0.01 | 0.004 |  |
| Homogeneity | 0.06 | 0.05 | 0.04 | 0.06 | 0.08 | 0.07 | 0.07 |  |
| GLRL |  |  |  |  |  |  |  |  |
| SRE | 0.04 | 0.04 | 0.03 | 0.04 | 0.06 | 0.05 | 0.05 |  |
| LRE | 0.04 | 0.04 | 0.03 | 0.05 | 0.06 | 0.05 | 0.05 |  |
| GLN | 0.04 | 0.04 | 0.03 | 0.05 | 0.06 | 0.05 | 0.05 |  |
| RLN | 0.04 | 0.04 | 0.03 | 0.05 | 0.06 | 0.05 | 0.05 |  |
| RP | 47.2 | 24.4 | 29.2 | 44.7 | 55.3 | 55.0 | 66.2 |  |
| LGRE | 48.0 | 25.0 | 29.3 | 45.1 | 56.0 | 55.9 | 66.9 |  |
| HGRE | 46.9 | 24.3 | 30.3 | 44.3 | 54.1 | 54.3 | 65.3 |  |
| SRLGE | 48.0 | 24.8 | 28.7 | 45.5 | 56.8 | 56.5 | 67.8 |  |
| SRHGE | 1268.9 | 880.8 | 631.6 | 1357.0 | 1699.4 | 1679.9 | 1735.8 |  |
| LRLGE | 1405.1 | 1072.3 | 815.1 | 1480.3 | 1876.0 | 1854.8 | 1914.6 |  |
| LRHGE | 1262.8 | 931.9 | 693.0 | 1310.0 | 1683.1 | 1661.7 | 1738.3 |  |

| **Supplement Table 1. Texture parameters: Standard deviation by flip angle, continued.** | | | | | | | |
| --- | --- | --- | --- | --- | --- | --- | --- |
|  | 2 (n=36) | 5 (n=36) | 10 (n=36) | 15 (n=36) | 20 (n=36) | 25 (n=36) | 30 (n=44) |
| Law's features |  |  |  |  |  |  |  |
| L1 | 7609.3 | 7892.3 | 10795.5 | 7716.7 | 7807.2 | 8456.5 | 8258.3 |
| L2 | 744.4 | 704.1 | 1010.5 | 772.2 | 1877.7 | 1929.9 | 1211.3 |
| L3 | 277.2 | 303.5 | 586.2 | 329.8 | 546.0 | 565.5 | 380.0 |
| L4 | 1809.6 | 1850.9 | 2572.3 | 1808.9 | 2239.3 | 2421.7 | 2147.6 |
| L5 | 340.8 | 353.8 | 403.5 | 453.1 | 881.8 | 891.3 | 427.0 |
| L6 | 281.1 | 272.1 | 327.4 | 366.9 | 681.5 | 682.7 | 308.3 |
| L7 | 132.5 | 152.2 | 339.6 | 182.4 | 337.5 | 334.5 | 170.2 |
| L8 | 854.2 | 822.4 | 884.3 | 990.9 | 1803.4 | 1829.8 | 872.1 |
| L9 | 1064.8 | 1098.6 | 1514.4 | 1102.7 | 1468.1 | 1603.7 | 1373.0 |
| GLGM |  |  |  |  |  |  |  |
| MGR | 3.1 | 2.6 | 2.7 | 3.4 | 3.9 | 4.1 | 4.0 |
| VGR | 2226.9 | 1930.7 | 1919.6 | 2432.5 | 2724.9 | 2872.3 | 2842.2 |
| Skewness | 1.1 | 0.94 | 0.88 | 1.2 | 1.3 | 1.5 | 1.6 |
| Kurtosis | 20.0 | 17.8 | 16.2 | 22.4 | 25.4 | 28.4 | 32.2 |
| Mean skewness | 0.58 | 0.41 | 0.26 | 0.56 | 0.72 | 0.71 | 0.75 |
| Mean kurtosis | 0.71 | 0.60 | 0.78 | 0.75 | 0.65 | 0.62 | 0.58 |
| Mean laws | 12832.8 | 13114.3 | 16293.3 | 13137.0 | 16769.2 | 17787.1 | 14279.6 |

Abbreviations: n=number of contoured slices; STD=standard deviation; STD5=5-neighborhood standard deviation; STD9=9-neighborhood standard deviation; IQR=indicates interquartile range; GLCM=gray-level co-occurrence matrix; GLRL=gray-level run length; SRLGE=short-run low gray-level emphasis; SRHGE=short-run high gray-level emphasis; GLGM=gray-level gradient matrix; SRE=short-run emphasis; LRE=long-run emphasis; GLN=gray-level nonuniformity; RLN=run-length nonuniformity; RP=run percentage; LGRE=low gray-level run emphasis; HGRE=high gray-level run emphasis; SRLGE=short-run low gray-level emphasis; SRHGE=short-run high gray-level emphasis; LRLGE=long-run low gray-level emphasis; LRHGE=long-run high gray-level emphasis; MGR=mean gradients; VGR=variance of gradients.
